# Omics Analysis of Chemoresistant Triple Negative Breast Cancer Cells Reveals Novel Metabolic Vulnerabilities

**DOI:** 10.3390/cells11172719

**Published:** 2022-08-31

**Authors:** Dimitris Kordias, Christina E. Kostara, Styliani Papadaki, John Verigos, Eleni Bairaktari, Angeliki Magklara

**Affiliations:** 1 Biomedical Research Institute-Foundation for Research and Technology, 45110 Ioannina, Greece; 2 Department of Clinical Chemistry, Faculty of Medicine, University of Ioannina, 45110 Ioannina, Greece; 3 Institute of Biosciences, University Research Center of Ioannina (URCI), 45110 Ioannina, Greece

**Keywords:** triple negative breast cancer, drug resistance, transcriptomics, metabolomics, lipidomics, MSMO1, myo-inositol, cholesterol biosynthesis

## Abstract

The emergence of drug resistance in cancer poses the greatest hurdle for successful therapeutic results and is associated with most cancer deaths. In triple negative breast cancer (TNBC), due to the lack of specific therapeutic targets, systemic chemotherapy is at the forefront of treatments, but it only benefits a fraction of patients because of the development of resistance. Cancer cells may possess an innate resistance to chemotherapeutic agents or develop new mechanisms of acquired resistance after long-term drug exposure. Such mechanisms involve an interplay between genetic, epigenetic and metabolic alterations that enable cancer cells to evade therapy. In this work, we generated and characterized a chemoresistant TNBC cell line to be used for the investigation of mechanisms that drive resistance to paclitaxel. Transcriptomic analysis highlighted the important role of metabolic-associated pathways in the resistant cells, prompting us to employ ^1^H-NMR to explore the metabolome and lipidome of these cells. We identified and described herein numerous metabolites and lipids that were significantly altered in the resistant cells. Integrated analysis of our omics data revealed MSMO1, an intermediate enzyme of cholesterol biosynthesis, as a novel mediator of chemoresistance in TNBC. Overall, our data provide a critical insight into the metabolic adaptations that accompany acquired resistance in TNBC and pinpoint potential new targets.

## 1. Introduction

One in eight women will develop breast cancer during her lifetime turning it into the most frequent tumor type in both sexes with >2.2 million new cases diagnosed in 2020 worldwide [1]; this number is expected to climb to >3 million per year by 2040 (https://gco.iarc.fr/tomorrow) (accessed on 1 July 2022). Despite significant advances in diagnosis and treatment, the death toll from breast cancer reached ~0.7 million in 2020 [1] and is predicted to rise to ~1 million per year by 2040 (https://gco.iarc.fr/tomorrow) (accessed on 1 July 2022). Even though novel promising therapeutic modalities have been introduced into clinical practice, conventional chemotherapy, including anthracyclines, alkylating agents and/or taxanes, remains the frontline treatment, especially for patients with triple negative breast cancer (TNBC), who lack therapeutic targets [2]. Most TNBC patients initially show a good response to chemotherapy, but the majority will present with relapse and metastasis 3–5 years after diagnosis as they become drug-resistant, and they will eventually die of the disease [3].

Metabolic reprogramming describes the alterations in the metabolic pathways of tumor cells, compared to their healthy counterparts, and is now considered one of the hallmarks of cancer [4], as it is essential for the support of the neoplastic state. Rapid production of ATP and intermediate biomolecules is necessary for the intense macromolecule biosynthesis occurring in the continuously dividing tumor cells and is ensured via their switching to a more glycolytic phenotype [4]. This metabolic reprogramming is believed to be triggered by mutations in oncogenes that affect the expression and activity of key metabolic enzymes, leading to dysregulation of the associated pathways [4].

Interestingly, further metabolic rewiring occurs in certain cancer cells upon drug exposure, as they adapt to the cytotoxic stress and develop mechanisms to evade it. For example, chemoresistant cancer cells exhibit higher glucose and glutamine uptake and increased catabolic rate of these nutrients, increased fatty acid oxidation and preference for certain amino acids, when compared to their drug-sensitive counterparts (reviewed in [5]). Analysis of the metabolomes of these two tumor populations may lead to the identification of metabolic traits in the resistant cells that could be potentially targeted and can resensitize these cells to drugs.

In the present work, we have developed and characterized a paclitaxel-resistant TNBC cell line to be used as an in vitro model for target identification and verification. Analysis of the transcriptomes of parental and drug-resistant cells revealed that the most significantly upregulated genes in the latter were the ones associated with metabolic pathways and especially with lipid metabolism. We, therefore, proceeded with the metabolomic and lipidomic profiling of the parental and paclitaxel-resistant cells. We identified numerous metabolites and lipids that were significantly altered. Among them, myo-inositol, a component of membrane lipids that is known to suppress tumor growth in vitro and in vivo, showed a marked decrease in the resistant cells. Furthermore, integrated analysis of our *omics* data led to the identification of *MSMO1*, a gene encoding for an intermediate enzyme of cholesterol biosynthesis, as a novel mediator of chemoresistance in TNBC. Thus, our work provides an important insight into the metabolic reprogramming that occurs in acquired resistance of cancer cells to paclitaxel and highlights potential targets for the development of new drugs that may be more effective against breast cancer.

## 2. Materials and Methods

### 2.1. Cell Culture and Generation of Paclitaxel-Resistant Cell Line

The SUM159 human TNBC cell line that belongs to the aggressive mesenchymal-like subtype [6] was a generous gift from Dr. Weinberg (Whitehead Institute, Boston, MA, USA). It was cultured in Ham’s F12 (LM-H1235/500, Biosera), supplemented with 5% fetal bovine serum (Ref 10437-028, Gibco), 1% penicillin/streptomycin (XC-A4122/100, Biosera), 5 μg/mL insulin (I9278, Sigma, St. Louis, MO, USA) and 1 μg/mL hydrocortisone (H0888, Sigma) in a humidified atmosphere of 5% CO_2_ at 37 °C. Cells were routinely passaged every 2 or 3 days and tested for mycoplasma. Paclitaxel-resistant SUM159 cells were generated by exposure to 25 cycles of a 2-day treatment with escalating doses (0.005 μM–1 μM) of paclitaxel (PATAXEL, Vianex S.A., Athens, Greece), followed by a recovery period in a drug-free medium.

### 2.2. Cell Proliferation Assay

Cell proliferation assay was performed using the Incucyte Zoom system (Essen BioScience, Hertfordshire, United Kingdom) and software, as per the manufacturer’s protocol. Briefly, the SUM159 parental and paclitaxel-resistant cells were seeded in triplicate into 96 well plates at low confluency, and the Incucyte Zoom live-cell imaging system was used to obtain phase contrast images of the cells every 6 h for a total of 72 h. Confluency was determined using the associated software and the increase in cell number per well was determined using the confluence readings at the beginning and end of the experiment. To evaluate the effect of paclitaxel, serial dilutions of the drug (0.001–5 μM) were added to the cells for 48 h, cell proliferation was estimated as described above and the GraphPad Prism 8.01 (San Diego, CA, USA) software was used to calculate the IC_50_ values.

### 2.3. MSMO1 Knock-Down

Two small interfering RNAs (siRNAs) (siMSMO1-1: 5′-GAACAGACUCUCAGUAUAAdTdT-3′ and siMSMO1-2: 5′-GCUGUGGAAUAUGUAGAUUdTdT-3′) previously published [7] were used for transient *MSMO1* knock-down and were transfected into cells using Lipofectamine™ RNAiMAX Transfection Reagent (ThermoFisher Scientific, Waltham, MA, USA), according to manufacturer’s instructions. A scrambled siRNA was used as a negative control.

### 2.4. Immunofluorescence Analysis

SUM159 parental and paclitaxel-resistant breast cancer cells were seeded on coverslips and the next morning they were treated with 0.5 μM of paclitaxel for 48 h. Subsequently, the cells were washed with PBS, fixed with 4% PFA and incubated with a primary antibody against tubulin (1:500) (Developmental Studies Hybridoma Bank (DSHB)-E7) for 1 h at room temperature (RT), followed by a secondary antibody (Goat anti-Mouse IgG Alexa Fluor 488, 1:400, cat. No. A-1100, ThermoFisher Scientific, Waltham, MA, USA) for 45 min at RT. TOPRO-3 (cat. No. T3605, Invitrogen, ThermoFisher Scientific, Waltham, MA, USA) and was used for DNA staining. A Leica SP5 confocal microscope was used to image the specimens. All images were obtained using the same parameters (PMTs and offset) and were processed in the same manner.

### 2.5. Quantification of Intracellular Doxorubicin by Fluorescence-Activated Cell Sorting (FACS)

SUM159 parental and paclitaxel-resistant cells were seeded in 12 well plates and the next day they were treated with different concentrations of doxorubicin (Adriblastina, Hydrochloride 10 mg/5 mL VIAL, Pfizer) for 6 h. Subsequently, cells were collected, centrifuged (1500 RPM, 5 min, RT), resuspended in 100 μL PBS and analyzed using flow cytometry on a BD FACS Aria II instrument (BD Biosciences, San Jose, CA, USA). Cells were monitored based on the intrinsic fluorescence emitted by doxorubicin using the phycoerythrin filter.

### 2.6. RNA Extraction, cDNA Synthesis and q-RT-PCR

For total RNA extraction the RNeasy Kit (Qiagen, Germantown, MD, USA) was used. Total RNA concentration and purity was measured using the NanoDropTM 2000 (ThermoFisher Scientific, Waltham, MA, USA). For cDNA preparation and q-RT-PCR experiments the PrimeScript 1st strand cDNA Synthesis Kit (TAKARA, Kusatsu, Shiga, Japan) and the KAPA SYBR^®^ FAST qPCR Kit Master Mix (2x) (cat. No. KK4602, KAPA BIOSYSTEMS) were used, respectively.

### 2.7. RNA-Sequencing (RNA-seq) and Bioinformatic Analysis

RNA-seq libraries were prepared using the TruSeq RNA v2 kit (Illumina, San Diego, CA, USA) from 1 μg of total RNA. The libraries were checked with the Agilent bioanalyzer (DNA1000 chip) (Agilent, Santa Clara, CA, USA), quantitated with the qubit HS spectrophotometric method and pooled in equimolar amounts for sequencing. Approximately 25 million, 75-bp long, single-end reads were generated for each sample on an Illumina NextSeq500 sequencer. For each sample, two biologically independent replicates were sequenced yielding highly similar results.

Quality Control was performed with the fastq raw data file using the “FASTQC” software (GPL v.3, Babraham Institute, Cambridge, UK). Normalization was performed with the estimate size factor function followed by Differentially Expressed Genes Analysis. The count files were used as input for DESeq2 (Bioconductor version: Release (3.13) [8]) for the identification of DEGs between paclitaxel-resistant and parental cells with a statistically significant cut-off value of *p*-adjust < 0.01. Additional cut-off criteria were set as fold-change ≥ 2 and number of reads > 10. Gene ontology (GO) analysis for the DEGs was performed using the Database for Annotation, Visualization and Integrated Discovery (David) (https://david.ncifcrf.gov/) (accessed in March 2022). Only categories with a *p*-value < 0.05 were further analyzed.

### 2.8. Metabolite and Lipid Extraction

The SUM159 parental and paclitaxel-resistant cells were cultured as described above. Cells were routinely passaged every 2 or 3 days. For the extraction of metabolites and lipids of passage 3 cells, a modified protocol described by Teng Q et al. was used [9]. Briefly, a mixture of methanol, chloroform and water in a ratio of 1:1:0.33 (*v/v/v*) was used for the generation of a dual-phase procedure. Approximately 15 million cells of each cell line were rinsed once using warm PBS and subsequently, cells were quenched using 6 mL of cold HPLC-grade methanol (−80 °C) and were detached using a cell scraper. Cells were collected in a glass tube and 3 mL of cold chloroform (−80 °C) were added to the tube. Cells were vigorously vortexed, incubated on ice for 15 min and sonicated 3 times for 30 s each round using a sonication probe in 30% amplitude. Addition of 3 mL of chloroform and vigorous vortex followed. Finally, 2 mL of water were added in the tubes and cells were vortexed and centrifuged at 1000× *g* for 15 min at 4 °C. After centrifugation, two phases were generated. The methanol/water phase contained the polar metabolites, while the chloroform phase contained non-polar lipid molecules. Proteins and other macromolecules were trapped in the interphase. After collecting each phase separately, solvents were evaporated under gentle N_2_ flow. Metabolites and lipids were stored at −80 °C until NMR analysis.

### 2.9. ^1^H NMR Spectroscopy

All ^1^H NMR experiments were carried out on a Bruker Avance DRX NMR spectrometer (NMR Center, University of Ioannina) operating at 500 MHz (proton resonance frequency).

Intracellular metabolites of breast cancer cells: A Bruker standard 1D Nuclear Overhauser Enhancement Spectroscopy (NOESY) presaturation pulse sequence (RD-90°-t1-90°-tm-90°-FID), with a relaxation delay of 4 s and a mixing time of 0.01 s was used for all NMR experiments to suppress the water signal at 300 K. For each sample, the ^1^H NMR spectrum was collected with 128 scans into 64 K computer data points with a spectral width of 10,000 Hz and an acquisition time of 3.28 s. The free induction decays (FIDs) were multiplied with an exponential line-broadening factor of 0.3 Hz prior to Fourier transformation. The phase and baseline of NMR spectra were manually corrected by applying a simple polynomial curve fit with the TopSpin software package version 4.0.6 (Bruker Biospin, Rheinstetten, Germany) and the chemical shifts were referenced to TSP (δ = 0.00 ppm).

Lipids of breast cancer cells: The dried lipid extracts of parental and chemoresistant breast cancer cells were redissolved in a 500-microliter mixture of deuterated methanol/chloroform (2:1, *v/v*). A “zgpr” Bruker pulse program was applied with the parameters as follows: 64 scans, a 90° pulse, a relaxation delay of 4 s and a 5000-Hertz spectral width at 298 K. All FIDs were multiplied by an exponential weighting function corresponding to 0.3 Hz line-broadening factor and Fourier transformed into 32 K data points. The acquired ^1^H NMR spectra were manually corrected for phase and baseline distortions using TopSpin software package version 4.0.6 (Bruker Biospin, Rheinstetten, Germany). Quantification of the lipids was based on the integration of selected signals in the proton NMR spectrum, corrected for the number of protons and then normalized with respect to the signal area from the cholesterol C18 methyl group. The lipid composition was expressed as percentages of the total lipids of breast cancer cells.

### 2.10. Targeted Metabolomic Profiling and Metabolomic Data Analysis

The Chenomx NMR Suite 8.4 profiler (Chenomx, Edmonton, AB, Canada) and the Human Metabolome Database (http://www.hmdb.ca) (accessed January–May 2022) were used for the identification of metabolites. The 500-Megahertz reference library of the Chenomx NMR Suite software was used for the quantification of metabolites, and the values of the metabolite concentration were presented in μM.

One-factor statistical analysis, enrichment pathway and joint-pathway analysis were performed using MetaboAnalyst 5.0 (https://www.metaboanalyst.ca/) (accessed January–June 2022).

The multivariate supervised method Partial-Least-Squares-Discriminant Analysis (PLS-DA) [10] was used to construct a pattern recognition model for the parental and resistant cells based on the 23 metabolites identified and quantified. Data were normalized by median, while the data scaling was set at mean-centered and divided by the standard deviation of each variable prior to analysis. The PLS-DA analysis eliminated the uncorrelated systematic variation and described the maximum separation between different groups. The most important metabolites for the separation between the two groups were ranked using the variable importance in projection (VIP) score. A 10-fold cross-validation (10-fold CV) with Q^2^ as measure performance was used to select the optimum number of components for classification. The Q^2^ value depicts a prediction error having 1 as the optimal value, while a negative value of Q^2^ reflects an unreliable predictive model. For further validation of the model, a permutation test was performed. Finally, a two-sample *t*-test was used to examine the statistically significant difference for each metabolite separately between the two groups.

Quantitative enrichment analysis was performed using the small molecule pathway database (SMPDB) and Kyoto Encyclopedia of Genes and Genomes (KEGG) pathways. Data were normalized before the analysis as described above.

For joint-pathway analysis, all quantified metabolites and only the upregulated DEGs in the paclitaxel-resistant cells were assessed, using the following parameters: metabolic pathways (integrated), enrichment analysis-hypergeometric test, topology measure-degree centrality and integration method-combined *p*-values (unweighted)”.

### 2.11. Statistical Analysis of Lipidomic Data

Univariate analysis: All data were expressed as mean value ± SD. Group comparisons were performed with *t*-test for normally distributed data using the SPPS software v.22.0 (IBM, Armonk, NY, USA). A *p*-value < 0.05 was considered to indicate statistical significance.

Multivariate analysis: Unsupervised (principal component analysis, PCA) and supervised (orthogonal projections to latent structures discriminant analysis, OPLS-DA) multivariate techniques were used to construct a statistical model to extract specific lipidomic signatures of breast cancer cells associated with chemoresistance.

Prior to analysis, NMR spectra were divided into buckets using the AMIX 3.9 software (Bruker Biospin Corporation, Billerica, MA, USA). All data were normalized to the total spectrum area and mean-centered prior to multivariate data analysis, carried out with the SIMCA-P+ 14 software (Umetrics, Umea, Sweden). Initial exploration of the lipidomic data was performed with PCA to identify possible groupings, trends and potential outliers before supervised multivariate analysis using OPLS-DA. The OPLS-DA analysis eliminated the uncorrelated systematic variation and described the maximum separation based on class membership. The OPLS-DA scores plot was used to show observations lying outside the 0.95 Hotteling’s T2 ellipse and to detect any grouping trend or separation, whereas the OPLS-DA loading coefficient plot was used to show the contributions of all NMR spectral regions or variables (corresponding to lipid components) to the grouping trend or separation seen in the OPLS-DA scores plot. The performance of the OPLS-DA model was assessed by goodness-of-fit parameters R^2^ (R^2^X and R^2^Y) and Q^2^, related, respectively, to the explained and predicted variance calculated through 7-fold CV. Cross-validated analysis of variance (CV-ANOVA) was also used to assess the significance of the OPLS-DA model. When CV-ANOVA *p*-value was < 0.5, the OPLS-DA model was considered reliable.

## 3. Results

### 3.1. Establishment and Characterization of a Paclitaxel-Resistant TNBC Cell Line

**Figure 1:**
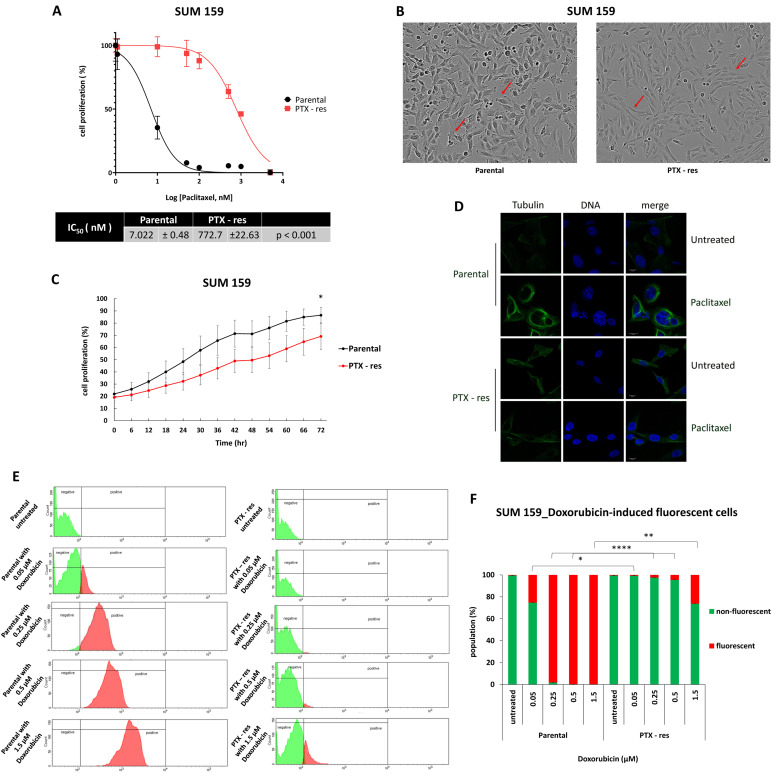
Establishment and characterization of SUM159 paclitaxel-resistant (PTX-res) cells. (**A**) The generated cell line (PTX-res) demonstrates > 100-fold resistance, compared to the parental cells, when treated with paclitaxel, as shown by their IC_50_ values. (**B**) The establishment of the drug-resistant phenotype in SUM159 cells is associated with alterations in cellular morphology. (**C**) Growth kinetics assay for SUM159 parental and PTX-res cells grown in culture for three days. (**D**) Immunofluorescence staining for SUM159 parental and PTX-res cells with an antibody against tubulin (green) or TORPO3 (blue). (**E**) FACS analysis in SUM159 parental and PTX-res cells for intracellular uptake of doxorubicin (red fluorescence) after a 6-hour incubation with different drug concentrations. (**F**) Quantification of FACS analysis presented in (**E**). Error bars indicate the SEM of biological replicates (n = 3). *: *p* < 0.05, **: *p* < 0.01, ****: *p* < 0.0001.

To identify distinct traits of paclitaxel-resistant TNBC that can be used as potential targets, we sought to develop an in vitro model by repeatedly exposing the SUM159 breast cancer cells to escalating doses of the drug (0.005–1 μM). The cytotoxic effect of paclitaxel was monitored by employing real-time imaging using the Incucyte ZOOM system. The chemoresistance of the cells under treatment was examined periodically by comparing their IC_50_ value with that of the parental cells after 48 h of drug exposure. Eventually, the generated cell line was >100-fold resistant to paclitaxel compared to the parental one, with their IC_50_ values being 772 nM and 7 nM, respectively (Figure 1A). The morphology of the paclitaxel-resistant (called PTX-res hereafter) cells was different than that of the parental ones (Figure 1Β) with the former presenting a more mesenchymal phenotype, a common feature of chemoresistant cells [11,12]. Cell growth assays showed that the PTX-res cells also had a significantly slower proliferation rate compared to the parental ones (*p* < 0.05) (Figure 1C), an adaptation frequently developed by cancer cells to evade the cytotoxicity of chemotherapeutic drugs targeting the rapidly proliferating cells [13].

It is well-established that paclitaxel binds to the β-tubulin subunit of microtubules causing excessive stabilization, thus preventing normal formation of the mitotic spindle during cell division and eventually leading to mitotic arrest and cell death [14]. Immunostaining with an antibody against β-tubulin showed a normal distribution of microtubules in the cytoplasm both of the untreated parental and PTX-res cells (Figure 1D). Administration of paclitaxel led to the rearrangement of microtubules and formation of bundle-shaped, static structures in the cytoplasm of the parental cells, as well as to the appearance of micronuclei (Figure 1D), which is one of the outcomes of abnormal mitosis (reviewed in [15]). In contrast, the drug did not induce any changes in the microtubule organization of the PTX-res cells (Figure 1D), confirming that they had developed mechanisms that allowed them to evade its deleterious effects.

A common mechanism of acquired chemoresistance is the overexpression of ABC membrane transporters that mediate an increased drug efflux, thus restricting cell exposure to cytotoxic agents [16]. To investigate whether such a mechanism contributes to the paclitaxel resistance of our cell line, we took advantage of its cross-resistance to doxorubicin (Supplementary Figure S1A); this drug possesses intrinsic fluorescence that can be used in fluorescence-activated cell sorting (FACS) experiments to monitor its intracellular levels [17]. Parental and PTX-res cells were subjected to FACS analysis after exposure to increasing doses of doxorubicin and the results are presented in Figure 1E. Upon administration of 0.05 μΜ, 0.25 μΜ and 0.5 μM doxorubicin, almost all the PTX-res cells appeared negative for drug intake, while there was a dose-dependent increase in the percentage of the parental cells that stained positive (25.13%, 98.07% and 99.97%, respectively) (Figure 1F). Even at the highest drug concentration (1.5 μΜ), only ~18% of the resistant cells were positive for doxorubicin, while all the parental cells showed high intracellular levels of the drug (Figure 1F). Consequently, it is safe to assume that one of the mechanisms that the PTX-res cells had developed to survive the toxic levels of paclitaxel was the increased expression of multidrug efflux pumps. This assumption was verified by transcriptome analysis, as it is described in the next section.

Overall, we have established and characterized a new paclitaxel-resistant TNBC cell line that displays several of the “classic” characteristics of acquired chemoresistance, including a mesenchymal phenotype, a slower proliferation rate and an improved capacity for drug outflow from the cells. Therefore, this cell line can be used as a valid in vitro model to discover and study novel mechanisms that can be potentially targeted to alleviate resistance to paclitaxel in TNBC.

### 3.2. Transcriptomic Analysis of the SUM159 Parental and PTX-Res Cells

#### 3.2.1. Identification of Differentially Expressed Genes (DEGs)

**Figure 2:**
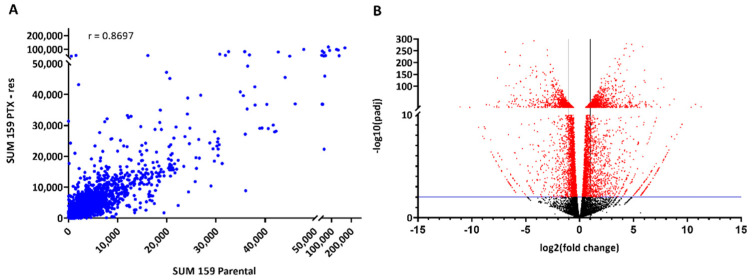
Transcriptomic analysis of SUM159 parental and PTX-res cells. (**A**) Scatter plot for the expression levels of the 27,967 genes analyzed by RNA-seq (presented as average number of reads). (**B**) Volcano plot (*p*-value vs. log2fold change of gene expression) of the 27,967 genes that were analyzed. Differentially expressed genes (DEGs) between parental and resistant cells are depicted in red, while the blue line indicates the cut-off of statistical significance (*p*-adjust value < 0.01). r: Pearson correlation coefficient.

To gain some insight into the gene networks involved in paclitaxel resistance in TNBC, total RNA from SUM159 parental and PTX-res cells was isolated and analyzed by RNA-seq. In total, we analyzed 27,967 genes that mostly exhibited a similar expression pattern between the two cell lines (Pearson correlation coefficient r = 0.8697), as expected (Figure 2A). Significant differences in the transcriptomic profiles of the two cell lines were determined by bioinformatic analysis using DESeq2 that yielded a total of 3184 DEGs (see Supplementary Table S1); 1802 DEGs were upregulated and 1382 were downregulated in the PTX-res cell line (Figure 2B).

A close inspection of our data revealed that several very highly expressed genes, such as *ABCB1*, *ABCB4*, *CROT*, *TP53TG1*, *DBF4*, *SLC25A40* and *SRI* (Supplementary Table S1), were located at the same region in chromosome 7. According to previously published studies, the amplification of the chromosome 7q21 region in tumors and cell lines after multiple rounds of drug treatment is a frequent event that leads to overexpression of the resident genes, including the aforementioned ones (reviewed in [18]). Notably, all these genes have been linked before to drug-resistance and/or tumorigenesis (reviewed in [18]).

Other DEGs that were upregulated in the PTX-res cells (Supplementary Table S1) included the β-tubulin encoding gene *TUBB3*, which has been associated with paclitaxel resistance [19] and *FZD2*, a member of the Wnt receptor Frizzled family, which has been shown to endow breast cancer cells with drug resistance [20]. On the other hand, the downregulated genes in the PTX-res cell line (Supplementary Table S1) included genes of which high expression levels are associated with a milder phenotype, such as *GATA3* and *CADM1*. The transcription factor GATA3 is overexpressed in lower grade breast tumors with a better prognosis, while its low levels are correlated with larger tumors [21,22]. Similarly, a lack of expression of the cell adhesion molecule *CADM1* is associated with an advanced tumor stage, suggesting that inactivation of *CADM1* promotes breast cancer development [23,24].

The above data, taken together, validate our transcriptomic analysis and further support the SUM159 PTX-res cell line as a sound model for extracting novel information for targeting chemoresistance in TNBC.

#### 3.2.2. Gene Ontology (GO) Analysis Reveals Cholesterol Biosynthesis as an Important Process in SUM159 PTX-Res Cells

To further investigate the biological significance of our data and uncover pathways that are involved in paclitaxel resistance, we performed GO analysis for the DEGs using the DAVID Functional Annotation Bioinformatics Microarray Analysis. We focused on the upregulated DEGs, since our main goal was to identify genes whose protein products could be potentially blocked or inhibited yielding a less resistant phenotype.

**Figure 3:**
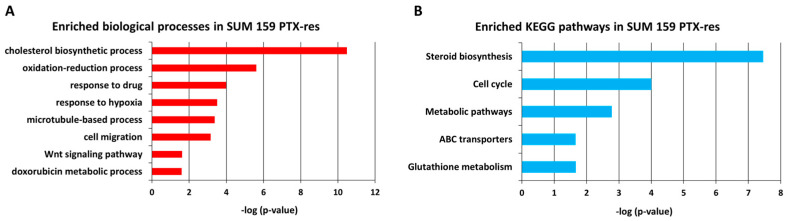
Gene Ontology (GO) analysis of SUM159 paclitaxel-resistant (PTX-res) cells (**A**). The most significantly enriched biological processes for the upregulated DEGs resulting from David analysis. (**B**) The most significantly enriched KEGG pathways for the upregulated DEGs resulting from David analysis.

Figure 3A illustrates the most significantly enriched biological processes in the SUM159 PTX-res cells. Notably, “cholesterol biosynthesis’’ is the process that tops the list. Cholesterol is an important component of the cell membrane and the precursor molecule of steroids. Recent studies have highlighted the accumulation of cholesterol in malignant tissues as a feature of cancer cells [25] and the involvement of cholesterol metabolic pathways in cancer cell resistance [26]. The other significantly enriched biological processes have also been associated with drug resistance before. It has been reported that redox homeostasis is involved in drug resistance in breast [27,28] and lung cancer [29]. As it was noted in the previous section, the overexpression of ATP-dependent efflux pumps, such as *ABCB1*, *ABCB4* and *ABCG2*, is a common phenomenon in drug-resistant cancers [30,31,32] and these genes were included in the “response to drug’’ process in our data (Figure 3A). Overexpression of hypoxia-related genes has been associated with poorer prognosis and drug resistance in breast cancer [33]. Increased expression levels of different classes of tubulin have been described as a mechanism of paclitaxel resistance in breast, ovarian and lung cancer [34,35,36]; such genes were identified in the “microtubule-based” process (Figure 3A), suggesting that this is one of the resistance mechanisms developed in our cell line. Several studies have demonstrated that drug treatment triggers the migration of cancer cells and that chemoresistant cells display a higher migration potential [37,38,39] explaining the appearance of the “cell migration” process in the upregulated DGEs in the PTX-res cells. Deregulation of the “Wnt signaling pathway’’ has been linked before to the development of resistance to anti-cancer agents leading to poor overall survival in TNBC [40]. Finally, the enriched process of “Doxorubicin metabolism’’ confirms the cross-resistance of PTX-res cells to doxorubicin, which we experimentally documented (Supplementary Figure S1A). 

In summary, the biological processes found enriched in the upregulated DEGs in the PTX-res cells are in accordance with previous studies reporting on properties of chemoresistant cells and further support the validity of our paclitaxel-resistant model.

We also performed KEGG pathway analysis for the upregulated DEGs and the results revealed fourteen significantly enriched pathways in the PTX-res cells with the top five presented in Figure 3B. This analysis confirmed “cholesterol/steroid biosynthesis” as the most significantly enriched process and also revealed other metabolism-associated pathways, in agreement with the prevailing concept that altered metabolism is one of the hallmarks of drug resistance in cancer [41].

The above data led us to study further the SUM159 parental and PTX-res cells seeking to verify that the transcriptomic discrepancies are also reflected on the metabolomic and lipidomic profiles of the cells.

### 3.3. Metabolomic Analysis of the SUM159 Parental and PTX-Res Cells

#### 3.3.1. Metabolite Quantification

**Figure 4:**
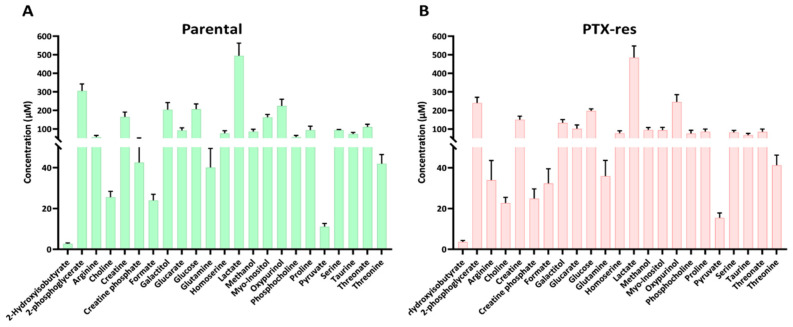
Quantification of metabolites extracted from the SUM159 parental and PTX-res cells. Extraction of metabolites using a two-phase process and metabolite quantification using the Chenomx software were conducted. Twenty-three metabolites were detected in both cell lines with high confidence. (**A**) The concentrations of the 23 metabolites in parental cells ranged from 28 ± 0.38 to 495 ± 67 μM. (**B**) The concentrations of the 23 metabolites in the PTX-res cells ranged from 3.6 ± 0.78 to 485 ± 63 μM. Error bars indicate the SEM of biological replicates (n = 6).

NMR spectra from SUM159 parental and PTX-res cells were processed using the Chenomx software. Metabolites that showed great variation among independent experiments or overlapped with other peaks in the spectra, making their identification and quantification questionable, were excluded from further analysis. In total, twenty-three metabolites were identified with high confidence and were quantified in both cell lines (Figure 4). The concentration of the 23 metabolites ranged widely from 2.8 ± 0.38 to 495 ± 67 μM in the parental (Figure 4A) and from 3.6 ± 0.78 to 485 ± 63 μM in the PTX-res cell line (Figure 4B). Several amino acids were detected in both cell lines, including arginine, glutamine, proline and threonine, ranging between 40–95 μM in the parental and between 33–85 μM in the PTX-res cells. Lactate, the main product of glycolysis, appeared to be the most abundant metabolite in both cell lines, while glucose, the main energy fuel of the cells, was detected in similar levels (208 ± 27 μM for parental and 198 ± 11 μM for PTX-res cells).

Nonetheless, several metabolites (arginine, creatine phosphate, myo-inositol and phosphocholine) appeared to vary considerably between the two cell lines (Supplementary Figure S2), which prompted us to proceed with a more systematic analysis and comparison of their overall metabolic profiles.

#### 3.3.2. Metabolic Profile Analysis Reveals Significant Differences between the SUM159 Parental and PTX-Res Cells

**Figure 5:**
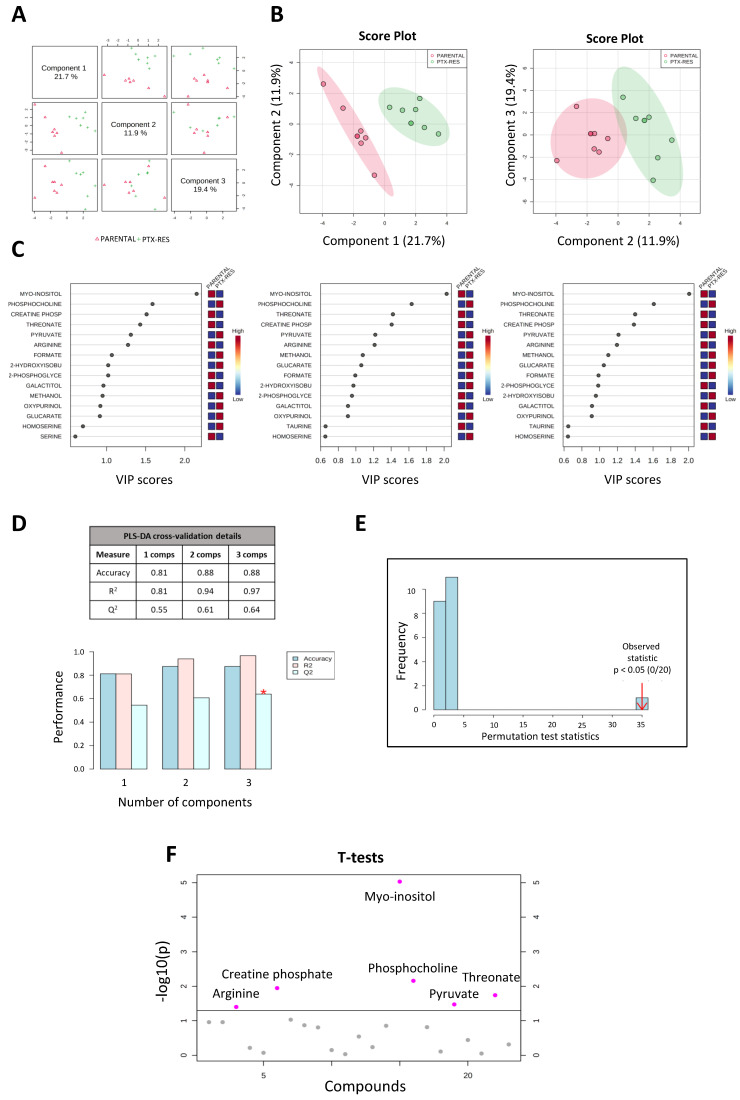
Partial Least Squares-Discriminate Analysis (PLS-DA) using MetaboAnalyst for SUM159 parental and PTX-res cells. (**A**) Pairwise score plot for the top three components. (**B**) Two-dimensional score plots for components 1 and 2 (left) and for components 1 and 3 (right), clearly showing the separation for the overall metabolic profiles of the two cell lines. (**C**) The 15 most important metabolites for group separation ranked by the variable importance in projection (VIP) score for each of the three components. The colored boxes indicate the relative concentrations of the corresponding metabolite in each group under study, with red indicating high and blue indicating low concentrations. Myo-inositol exhibits the highest VIP score. (**D**) A 10-fold cross-validation (10-fold CV) with Q^2^ as measure performance for the estimation of the predictive ability of the model. The R^2^ and Q^2^ values are presented in the table. (**E**) Permutation test showing a *p*-value < 0.05 for further validation of the model. The number of permutations was set at 20. (**F**) *t*-test using a *p*-value threshold of 0.05 was used for finding the statistically significant metabolites, with myo-inositol being the most significant one. * *p* < 0.05.

The supervised multivariable quantitative PLS-DA of the overall metabolic profiles of the SUM159 parental and the PTX-res cells was carried out to determine whether the two cell lines were metabolically distinct. This analysis revealed a sharp separation between their metabolic profiles, as shown in Figure 5. The pairwise score plot of the first three components and the 2D score plots of component 1 vs. 2 and component 1 vs. 3 are presented in Figure 5A,B, respectively. The 15 most important metabolites accounting for the separation of the metabolic profiles of the two cell lines were ranked using the Variable Importance in Projection (VIP) score. Metabolites with higher VIP scores are considered more relevant in group separation (Figure 5C). As shown in Figure 5C, myo-inositol, a component of membrane phospholipids that mediates cell signal transduction and participates in osmoregulation [42], is the most significant metabolite having a VIP score ≥2 in all three components. Phosphocholine, creatine phosphate, threonate, pyruvate, arginine, formate, 2-hydroxyisobutyrate and 2-phosphoglycerate also contributed strongly to the separation of the two metabolic profiles with a VIP score ≥1 in all three components (Figure 5C).

The validity and accuracy of the predicted model were assessed using a 10-fold cross-validation (CV) for the three components. In our data, the three components delivered a valid prediction model with Q^2^ being 0.64 and R^2^ being 0.97 (Figure 5D). Employment of the permutation test further validated the above predictive model. The histogram presented in Figure 5E shows the distribution of 20 sets of permutated samples with a *p*-value < 0.05. The bar on the right of the diagram represents the original sample, while the bars on the left represent the permutated samples. This test also confirms that there is a significant separation of the metabolic profiles of the two cell lines.

A two-sample *t*-test identified six metabolites with significantly different levels (*p*-value ≤ 0.05) between the two cell lines (Figure 5F). Myo-inositol exhibited reduced levels in the PTX-res cells, as did creatine phosphate, threonate and arginine. On the other hand, phosphocholine and pyruvate were increased in the resistant cells (see also next section).

It is noteworthy that most of these metabolites have been associated with cancer, where they seem to play complex roles [43,44,45,46]. Myo-inositol, which emerged as the most important compound in the separation of the two cell lines on the metabolic level, is described as a strong inhibitor of tumor initiation and progression [47]. Our data suggest that its reduced levels may be a marker of resistance to paclitaxel in TNBC.

#### 3.3.3. Metabolite Set Enrichment Analysis

Next, we performed metabolite enrichment analysis to determine whether there were biologically important sets of functionally related metabolites that were significantly enriched in our data. All the quantified metabolites were included in this analysis and the reference databases used were KEGG [48] as well as the small molecule pathway database (SMPDB) [49].

**Figure 6:**
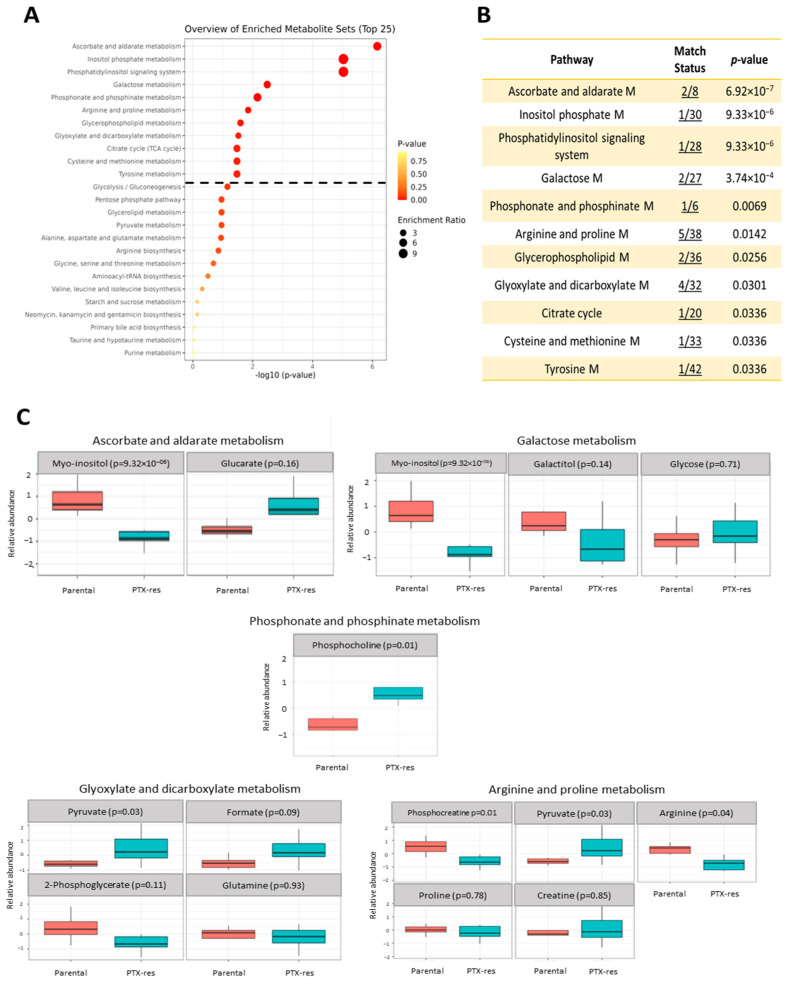
Metabolite enrichment analysis using MetaboAnalyst. (**A**) Quantitative enrichment analysis overview of the enriched metabolite sets derived from the quantified metabolites of parental and PTX-res cells based on the KEGG pathways library. The dashed line represents the cut-off for the significantly enriched metabolite sets in the PTX-res cells (*p* < 0.05 and FDR < 0.1). (**B**) Table that summarizes the KEGG metabolism-related pathways that were found enriched in the PTX-res cells. Match status indicates the number of metabolites found in our data vs. the number of metabolites in the library related to a specific pathway. (**C**) Concentration levels of the metabolites associated with the pathways presented in (**B**) in parental and PTX-res cells (M; metabolism).

Several KEGG metabolism-related pathways were found enriched in the PTX-res cells and the top 25 are presented as dot plots in Figure 6A. Eleven metabolite sets were found significantly enriched (*p* < 0.05 and FDR < 0.1) (Figure 6A,B). Figure 6C illustrates the concentration levels of the metabolites included in each set in the two cell lines. Μyo-inositol, which was decreased in the resistant cells, was present in the top four sets (Figure 6B,C). Phosphocholine, which was more abundant in the PTX-res cells (Figure 6C), was the only metabolite in our data associated with the “phosphonate and phosphinate metabolism” set (Figure 6B). The “arginine and proline metabolism” set was represented by five metabolites in our data (arginine, creatinine, proline, phosphocreatine and pyruvate) with arginine and phosphocreatine showing significantly lower and pyruvate showing significantly higher levels in the PTX-res cells (Figure 6C). Two metabolites, choline and phosphocholine, were associated with “glycerophospholipid metabolism” with the latter being significantly more abundant in the PTX-res cells (Figure 6C). Finally, out of the four metabolites associated with the metabolic set “glyoxylate and dicarboxylate metabolism”, only pyruvate showed significantly higher levels in the PTX-res cells (Figure 6C). Similar metabolite sets were significantly enriched (*p* < 0.05) in the resistant cells, when the analysis was conducted using the SMPDB (Supplementary Figure S3).

Overall, the above analysis revealed several pathways that contributed to the distinction between the metabolic profiles of the two cell lines. Next, we proceeded with an integrated analysis of the transcriptomic and metabolomic data to identify DEGs that play an important role in the altered metabolism of the PTX-res cells.

#### 3.3.4. Joint-Pathway Analysis

**Figure 7:**
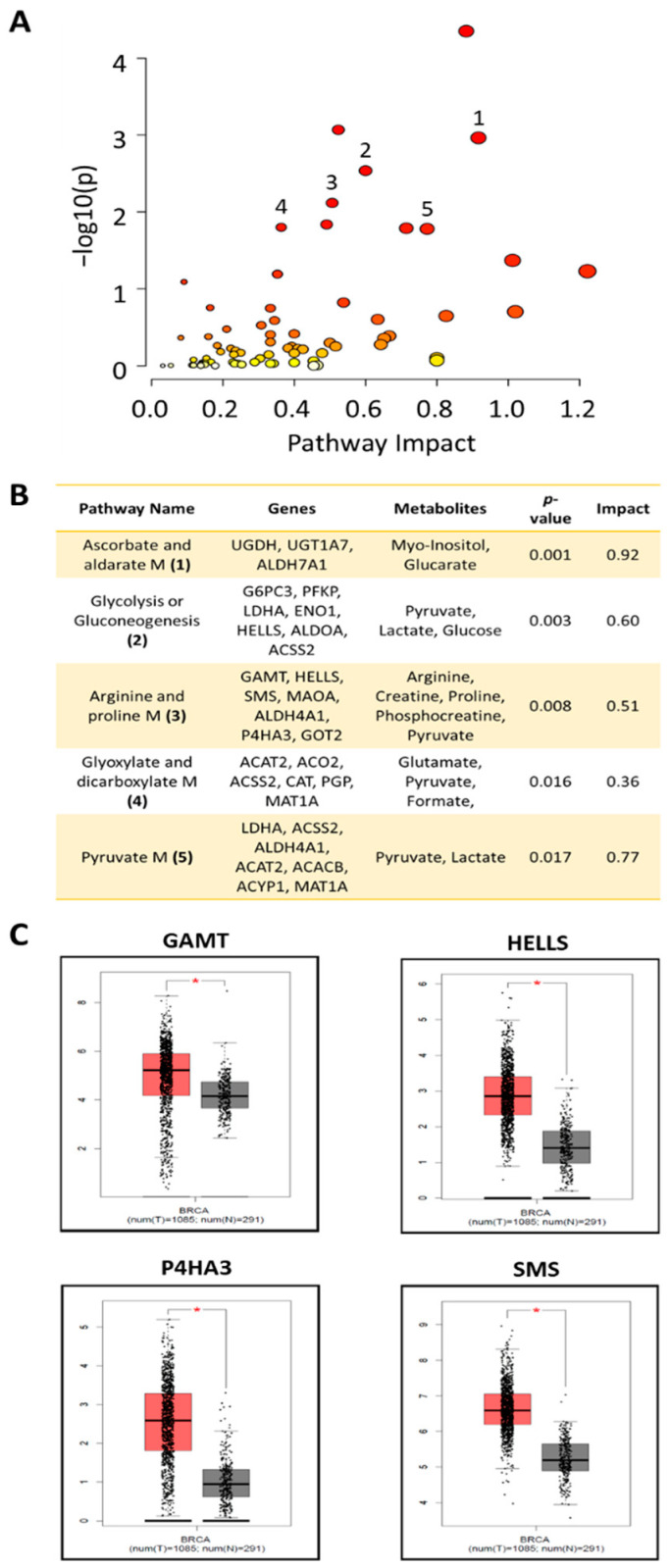
Joint-Pathway Analysis using MetaboAnalyst. (**A**) Overview of the matched pathways according to the *p*-values resulted from joint-pathway analysis of the upregulated DEGs and quantified metabolites. Dots illustrate all the pathways that were found enriched represented by upregulated DEGs and/or quantified metabolites. (**B**) The statistically significant enriched pathways (*p* < 0.05) represented by both upregulated DEGs and metabolites. Numbers indicated the respective dots in (**A**). Upregulated DEGs and quantified metabolites matching with each pathway are presented. (**C**) Genes associated with the “Arginine and proline M” and found statistically significant different between breast cancer tumors and normal samples in GEPIA database. M; metabolism/metabolites, G; genes. * *p* < 0.05.

Joint-pathway analysis was conducted using MetaboAnalyst [50] to find genes that are associated with the metabolic pathways that were found enriched in our data. All the quantified metabolites and the upregulated DEGs in the PTX-res cells were included in the analysis. An overview of the matched pathways from this analysis is shown in Figure 7A. There were five statistically significant enriched pathways, which were represented both by quantified metabolites and DEGs from our data, as shown in Figure 7B. “Arginine and proline metabolism” was the pathway that included the highest number of metabolites (five) and a high number of DEGs (seven). This prompted us to further explore the clinical significance of our findings using the GEPIA database (http://gepia.cancer-pku.cn/index.html) (accessed in April 2022). As shown in Figure 7C, four out of the seven upregulated DEGs in this metabolic set are significantly overexpressed in breast tumors compared to normal samples.

Hence, the joint analysis of our transcriptomics and metabolomics data pinpointed several metabolism-associated genes that are overexpressed in the PTX-res cells. Among them, the ones involved in the arginine–proline metabolism may be clinically significant in breast cancer.

### 3.4. Lipidomic Profile Analysis

#### 3.4.1. ^1^H NMR Spectra Analysis of the Lipid Extracts

Representative ^1^H NMR spectra of the lipid extracts from the parental and PTX-res cells are presented in Supplementary Figure S3. For visual comparison of the cell lipid composition, spectral intensity was normalized for the Tetramethylsilane (TMS) peak at 0.00 ppm. The lipid fraction of the PTX-res cells appeared to be enriched in free cholesterol (FC), choline phospholipids (PLs) [phosphatidylcholine (PC) and sphingomyelin (SM)] and total sphingolipids (SLs) and depleted of cholesterol esters (CE), lysophosphatidylcholine (LysoPC), plasmalogens, total ether glycerolipids (GLs), unsaturated fatty acids (UFA) and polyunsaturated fatty acids (PUFA) compared to that of the parental cells. Visual inspection did not reveal any marked differences between the two cell lines for signals attributed to phosphatidylethanolamine (PE), diacylglycerophospholipids (DAGPLs), triglycerides (TG), linoleic acid (LA) and fatty acid constituents such, as docosahexaenoic acid (DHA) and the sum of eicosapentaenoic and arachidonic acid (EPA + AA) (Supplementary Figure S4).

#### 3.4.2. Lipidomic Profiles of SUM159 Parental and PTX-Res Cells

We applied an untargeted and targeted lipidomic approach for the identification of differences in the lipid profiles of parental and PTX-res cells and for the quantitative alterations in the individual lipid classes.

**Figure 8:**
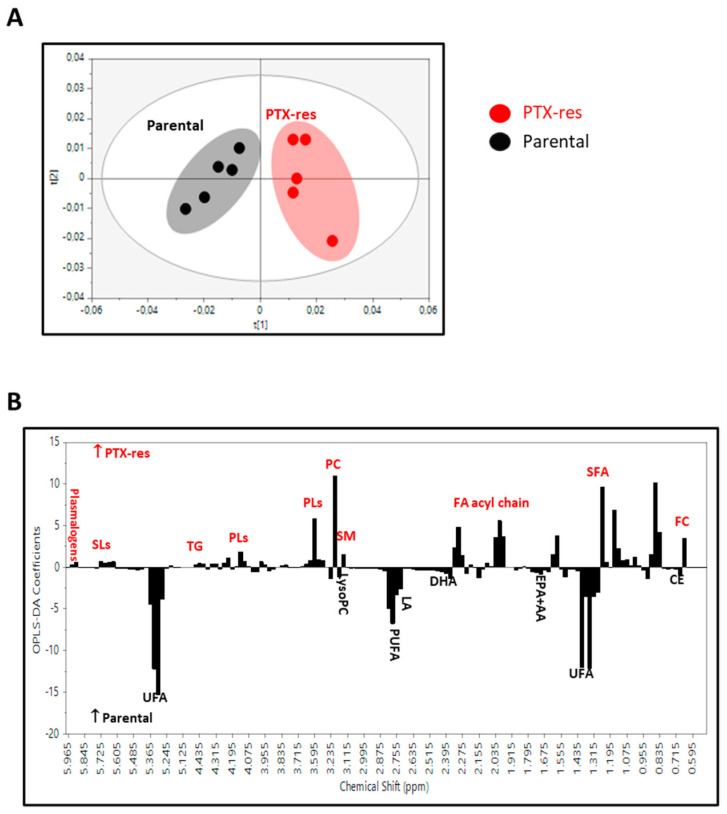
OPLS-DA score plot of the lipidomic data from (**A**) SUM159 PTX-res cells (red circles) and SUM159 parental cells (black circles); (**B**) the corresponding regression coefficient plot colored according to the correlation between the NMR lipidomic data and the group studied.

For the untargeted analysis, the buckets resulting from the division of the NMR spectra were initially examined by the unsupervised PCA to check the consistency and the quality of the data. The corresponding PCA scores plot (data not shown) revealed a separation trend between the PTX-res and parental cells and no distinct outliers were observed. In the OPLS-DA analysis, the score plot for the parental vs. PTX-res model (Figure 8A) showed a clear separation between the two groups with good goodness-of-fit and predictive power (R^2^X = 0.873, R^2^Y = 0.868, Q^2^ = 0.775 and *p* < 0.05). The analysis of the relative significance of each constituent in the groups’ separation, as illustrated in the loading coefficient plot (Figure 8B), revealed that the determining factors were the higher levels of PC, PLs and FC in the PTX-res cells, as well as SM, TG, SLs and plasmalogens, albeit with a lower significance, and the lower levels of CE. Furthermore, in the PTX-res cells, lipids were preferably esterified with saturated fatty acids (SFA) rather than UFA.

**Figure 9:**
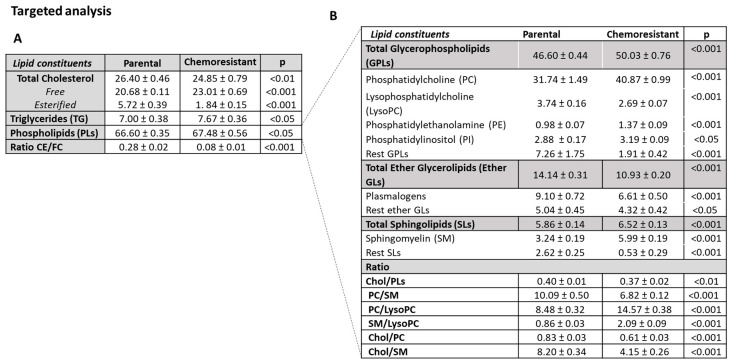
NMR-based compositional characteristics of (**A**) the major cellular membrane lipid classes and (**B**) phospholipids in PTX-res cells and parental cells. Values are expressed in percentages of total lipids (mol/100 mol of Total Lipid Content) and are means ± SD. Abbreviations: CE, Cholesterol Esters; DHA, Docosahexaenoic acid; EPA + AA, Eicosapentaenoic and Arachidonic acid; FA acyl chain, Fatty acid acyl chain, FC, Free cholesterol; LA, Linoleic acid; LysoPC, Lysophosphatidylcholine; PC, Phosphatidylcholine; PLs, Phospholipids; PUFA, Polyunsaturated fatty acids; SFA, Saturated fatty acids; SLs, Sphingolipids; SM, Sphingomyelin; TG, Triglycerides; UFA, Unsaturated fatty acids.

Thereafter, a quantitative targeted analysis of individual major and minor lipid constituents (Figure 9) and fatty acid pattern (Table 1) was performed. We quantified well-resolved signals in the NMR fingerprint attributed to protons of cholesterol in its free and esterified form, the headgroups and backbones of phospholipids and sphingolipids, the glycerol backbone, and fatty acids—All of them being esterified. It is worth noting that the results in the targeted analysis are totally consistent with those found in the untargeted multivariate analysis.

The percentage of total cholesterol (TC) was significantly lower in chemoresistant cells compared to parental due to the lower percentage of CE, whereas FC was significantly higher, resulting in a significant decrease in the CE/FC ratio (Figure 9). The significantly higher percentages of total glycerophospholipids (GPLs) and total SLs observed in PTX-res cells compared to parental have mainly caused the higher percentage of total PLs, while total ether GLs were significantly lower (Figure 9). PC, PE and PI presented a significant increase in PTX-res cells compared to parental, whereas LysoPC and the rest of GPLs were significantly lower. For the ether GLs, the percentages of the total content, plasmalogens and the rest ether GLs were significantly lower in PTX-res cells compared to parental. Finally, as seen in Figure 9B, the percentage of total SLs was higher in PTX-res cells compared to parental, mainly due to a significant increase in SM, while the percentage of the rest of SLs was significantly lower. The aforementioned alterations resulted in significantly higher molar ratios of PC/LysoPC and SM/LysoPC and lower ratios of TC/PLs, PC/SM, TC/PC and TC/SM in the PTX-res cells compared to parental. Thus, this analysis showed that PTX-res cells presented statistically significant differences in the percentages of all lipid classes (triglycerides, phospholipids and cholesterol in both free and esterified form) compared to parental.

Table 1 shows a comparison of the fatty acid composition of the lipid fraction from the two cell lines. The percentage of SFA was significantly higher in PTX-res cells compared to parental, whereas that of UFA was lower mainly due to individual PUFA such as LA, the sum of EPA + AA and DHA. Monounsaturated fatty acid content was not altered. These changes in the fatty acid pattern led subsequently to significantly higher ratios of saturated to polyunsaturated fatty acids in the PTX-res cells (Table 1) and thus, to a shift from the unsaturation toward saturation state that potentially affects the properties of cell membrane.

### 3.5. The Methylsterol Monooxygenase 1 (MSMO1) Gene Is Upregulated in the SUM159 PTX-Res Cells and Mediates Resistance to Paclitaxel

The fact that the analysis of the transcriptomics and lipidomics data converged to cholesterol and cholesterol biosynthesis (Figure 3A and Figure 9A) being upregulated in the PTX-res cells urged us to look into the associated genes more carefully. Notably, all genes encoding for the intermediate enzymes in the cholesterol biosynthesis pathway (shown in Supplementary Figure S5) were upregulated in the PTX-res cells (see Table S1) with the exception of *SC5DL*. The second most highly expressed gene was *MSMO1*, which is involved in several steps of cholesterol biosynthesis (shown in blue in Supplementary Figure S5) [51].

**Figure 10:**
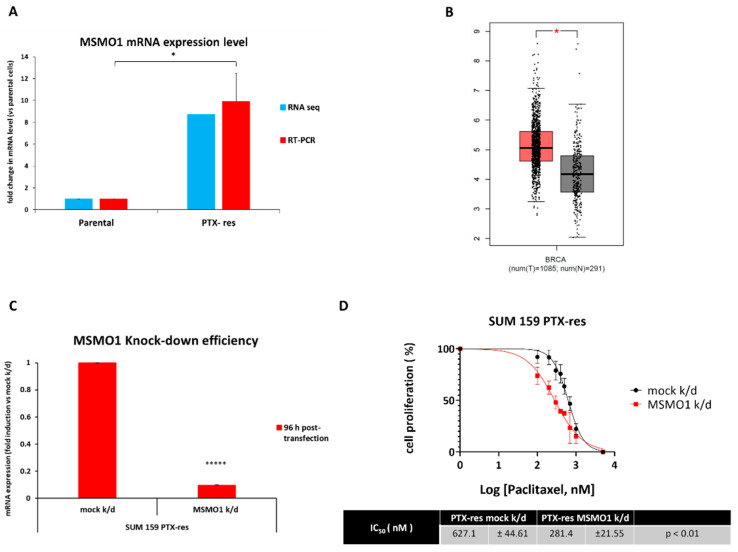
*MSMO1* is overexpressed and mediates paclitaxel resistance in SUM159 PTX-res cells. (**A**) *MSMO1* mRNA levels in SUM159 parental and PTX-res cells as determined by RNA-seq and RT-qPCR. (**B**) Box plot shows that *MSMO1* is overexpressed in breast tumors vs. normal tissue (data from GEPIA). (**C**) RT-PCR for *MSMO1* mRNA levels in SUM159 PTX-res cells after knock-down. (**D**) SUM159 PTX-res cells were knocked-down for *MSMO1* and then treated with different concentrations of paclitaxel. Cell confluency was measured using the Incucyte Zoom live-cell analysis system. The paclitaxel IC_50_ values of SUM159 PTX-res mock and SUM159 PTX-res *MSMO1* knock-down cells were calculated using Graphpad Prism version 8.01. Data from three independent experiments performed in triplicate are shown. Error bars represent the SEM of biological replicates. *: *p* < 0.05, *****: *p* < 0.00001.

Our RNA-seq data indicated that *MSMO1* mRNA expression levels were ~9-fold higher in the PTX-res cells compared to the parental ones, a finding that was also confirmed by RT-qPCR (Figure 10A). To determine whether *MSMO1* was clinically significant, we searched gene expression data from breast tumors using GEPIA and the results are presented in Figure 10B. The *MSMO1* mRNA levels are significantly increased in specimens from patients with breast cancer compared to normal breast tissue samples (Figure 10B).

To investigate the involvement of *MSMO1* in paclitaxel resistance, we knocked it down in SUM159 PTX-res cells (Figure 10C) and examined the effects by employing real-time imaging using the Incucyte ZOOM system (Figure 10D). The results showed that *MSMO1* knock-down significantly decreased the resistance of SUM159 PTX-res cells to paclitaxel with the IC_50_ value for the drug dropping from 627.1 nM to 281.4 nM (Figure 10D). These data strongly suggest that overexpression of *MSMO1* contributes to the acquired resistance to paclitaxel in the SUM159 PTX-res cells.

## 4. Discussion

Metabolic reprogramming is considered one of the hallmarks of cancer [4], and the analysis of the metabolic profile of cancer cells has recently gained attention as a diagnostic tool for cancer development and progression [52,53]. Metabolic adaptations can affect not only tumor growth and patient survival but can also mediate drug resistance [41] through mechanisms that have not been yet fully elucidated. Metabolomic and lipidomic analysis of chemoresistant cancer cells could lead to the identification of potential targets and the development of novel therapeutic approaches for cancer elimination. However, very few studies have investigated the metabolic profile of chemoresistant breast cancer cells [54]. In this work, we generated and characterized a paclitaxel-resistant TNBC cell line and investigated its transcriptome, metabolome and lipidome; integrated analysis of the *omics* data pinpointed several significant alterations compared to the parental cell line, suggesting that they may be exploited to target the resistant cells.

Metabolomic profiling using NMR is a wide-spread methodology, albeit with several limitations, especially concerning its sensitivity, which may result in lower concentrations of important metabolites or their masking by larger peaks [55]. Careful metabolite quantification of our spectra from the SUM159 parental and PTX-res cells yielded 23 metabolites with high confidence. We performed an untargeted analysis of all detected metabolites, which is especially useful when there is no *a priori* hypothesis for the determination of significantly perturbed metabolites [56]. This analysis revealed a clear separation of the metabolomic profiles between the two cell lines, which was mainly attributed to myo-inositol, but also to phosphocholine, creatine phosphate, threonate, pyruvate, arginine, formate, 2-hydroxyisobutyrate and 2-phosphoglycerate. Metabolite set enrichment analysis revealed that the most significantly enriched pathways in the PTX-res cells were “ascorbate and aldarate metabolism”, “inositol phosphate metabolism” and “phosphatidylinositol signaling system”; all three of these sets included myo-inositol, a carbocyclic sugar that is a precursor of inositol phospholipids and other derivatives.

Dysregulation of inositol metabolism has been associated with cancer [57], and myo-inositol has been found to exert multiple anti-cancer effects, including pro-apoptotic and anti-proliferative ones in various cancer types (reviewed in [47]). In a colorectal adenocarcinoma cell line, the inositol 3-phosphate synthase (ISYNA1) enzyme, which is essential for myo-inositol biosynthesis, was found to be a direct target of p53 [58]. It was suggested that p53 suppressed tumorigenesis by inducing myo-inositol biosynthesis. *ISYNA1* knock-down resulted in resistance to adriamycin treatment, proposing a role for myo-inositol biosynthesis in p53-mediated growth suppression [58]. However, there are some reports arguing against the involvement of p53 in the anti-cancer activity of myo-inositol (reviewed in [47]), hence, more conclusive studies are needed. Other groups have shown that myo-inositol inhibits the epithelial-to-mesenchymal program [59] and inflammatory factors [47] in breast cancer cells and that it induces the regression of different types of cancer (reviewed in [47]). Notably, in a prospective, randomized study, inositol hexakisphosphate and myo-inositol improved the responsiveness to adjuvant therapy in breast cancer patients and markedly increased their quality of life [60]. Therefore, mounting evidence suggests that myo-inositol exerts pleiotropic anti-cancer effects and, according to our data, its reduced levels could be a marker of paclitaxel resistance in TNBC. It is tempting to speculate that this metabolite mediates chemosensitivity, however, further mechanistic studies are needed to unequivocally establish such a property for myo-inositol and uncover the underlying cellular pathways.

The lipid composition of cancer cells is different from that of healthy cells, but it also varies between tumor types [61]. Furthermore, changes in the lipid metabolism of malignant cells have been recently reported to be associated with resistance to conventional chemotherapies (reviewed in [62]). We performed an NMR-based analysis of the lipidome of the PTX-res cells that revealed several aberrations in lipid-associated pathways, compared to the parental cells, including cholesterol and fatty acid biosynthesis, glycerophospholipid and sphingolipid metabolism. Both our transcriptomics and lipidomics data highlighted cholesterol biosynthesis as the main upregulated pathway in the PTX-res cells. These findings prompted us to look more closely to the genes associated with this pathway and we focused on *MSMO1* that encodes for an intermediate enzyme in cholesterol biosynthesis [51]. We confirmed that *MSMO1* was overexpressed in the PTX-res cells compared to the parental cells, while a GEPIA search revealed that its expression was significantly increased in breast cancer tissues compared to normal. During the course of the present study, a different group performed extracellular vesicle long RNA-sequencing in plasma from breast cancer patients and found that exMSMO1 mRNA level was a predictive biomarker for neoadjuvant chemotherapy of breast cancer [7]. Moreover, the silencing of *MSMO1* could enhance the sensitivity of breast cancer cells to paclitaxel and doxorubicin, presumably via the mTORC1 signaling pathway [7]. Along the same lines, we also found that knock-down of *MSMO1* significantly decreased the resistance of the PTX-res cells to paclitaxel, strongly suggesting that it could be used as a new potential therapeutic target for treating chemoresistant TNBC. Our *omics* data suggest that *MSMO1* overexpression leads to increased FC levels in the PTX-res cells. FC is located mainly in the cell membrane and is considered a major lipid modulator of its properties; due to the steroid ring system, increased levels of membrane cholesterol lead to decreased fluidity and increased rigidity. Cell membrane rigidity and permeability are strong determinants for the uptake and efficacy of chemotherapeutic agents and changes in these properties could contribute to cancer cells’ chemoresistance. Indeed, studies have shown that cells with higher membrane FC content tend to exhibit greater chemotherapy resistance compared to those with lower FC [61]. Consequently, our data are in agreement with an important role for high cholesterol levels in chemoresistance and suggest that *MSMO1* may be targeted to reduce the metabolite’s levels and improve response to chemotherapy. Future studies should aim to confirm a direct link between *MSMO1* overexpression, increased cholesterol levels and paclitaxel resistance.

Our results also demonstrated significant enrichment of the PTX-res cells with GPLs and SM, all of them being major lipid components of the cell membrane. Increased levels of PC and PE also lead to decreased membrane fluidity, resulting in increased resistance to drug influx [63]. Lysophospholipids mediate chemoresistance mainly through plasma membrane-independent mechanisms. It has been demonstrated that LysoPC mediates chemoresistance by protecting tumor cells from DNA damaging agents [64]. Finally, SM induces an increase in resistance to chemotherapy by promoting trafficking of the efflux pump MDR1 to plasma membrane lipid rafts [65].

The fatty acid pattern of lipids also regulates the physicochemical properties of the cell membrane. The shift of fatty acids from an unsaturated toward a saturated state that is observed in the PTX-res cells may promote a less flexible and more rigid membrane with the outcomes described above. Apart from their structural role, saturated and unsaturated lipids differ dramatically in terms of their susceptibility to peroxidation. Lipid peroxidation can lead to a form of oxidative stress-related cell death [66]. As saturated lipids are less susceptible to lipid peroxidation, PTX-res cells may be protected from such a death.

In conclusion, we used RNA-seq and NMR-based approaches to thoroughly characterize the transcriptome and metabolome of a new TNBC cell line resistant to paclitaxel. As our data underlined significant changes in lipid metabolism in the resistant cells, we undertook a lipidomics analysis that painted a completely different lipid profile for these cells compared to the parental ones. To our knowledge, this is the first report that integrates transcriptomics, metabolomics and lipidomics analysis for TNBC cells resistant to paclitaxel. The generated data should be valuable in the design of new therapeutic approaches that target the metabolome of these cells. As proof of concept, we have provided preliminary data that the cholesterol biosynthetic pathway may constitute a metabolic vulnerability of these cells that can be modulated by targeting *MSMO1*, which leads to significantly reduced drug resistance.

## Figures and Tables

**Table 1:** Fatty acid profile of the lipid extracts from PTX-res and parental cells. Values are expressed in percentages of total lipids (mol/100 mol of Total Fatty Acids) and are means ± SD.

	Parental	Chemoresistant	*p*-value
% Saturated	52.95 ± 0.60	55.69 ± 0.60	<0.05
% Unsaturated	47.05 ± 0.60	44.31 ± 2.17	<0.05
% Monounsaturated	4.19 ± 0.38	4.86 ± 0.51	NS
% Polyunsaturated	42.87 ± 0.80	39.45 ± 2.05	<0.05
Linoleic Acid Eicosapentaenoic + Arachidonic Acid Docosahexaenoic Acid	4.72 ± 0.11 15.58 ± 0.25 2.76 ± 0.06	3.93 ± 0.21 13.51 ± 1.14 2.29 ± 0.10	<0.001 <0.05 <0.001
**Ratio**			
Saturated/Unsaturated	1.13 ± 0.03	1.26 ± 0.11	NS
Saturated/Polyunsaturated	1.24 ± 0.04	1.42 ± 0.13	<0.05

## Data Availability

All raw and processed data RNA-seq files have been submitted to GEO (accession number GSE206242).

## Supplementary Materials

The following are available online at https://www.mdpi.com/article/10.3390/cells11172719/s1, Figure S1: PTX-res cell line presents cross-resistance to Doxorubicin; Figure S2: bar graph showing the significantly different metabolites quantified from SUM 159 parental and PTX-res cells. Figure S3: Metabolite enrichment analysis using MetaboAnalyst; Figure S4: typical 1H NMR 500 MHz spectra of lipid extracts from SUM159 PTX-res cells and SUM159 parental cells; Figure S5: schematic representation of the cholesterol biosynthesis pathway; Table S1: the 3184 differentially expressed genes (DEGs) between SUM159 parental and PTX-resistant cells identified by RNA-sequencing.
