# Supplementary material for: Omics Analysis of Chemoresistant Triple Negative Breast Cancer Cells Reveals Novel Metabolic Vulnerabilities
Source: Cells. 2022 Aug 31;11(17):2719. doi: 10.3390/cells11172719 (PMC9454761; doi:10.3390/cells11172719)
Supplement: Supplementary file 1 [file cells-11-02719-s001.zip › Supplementary Materials.pdf]

**Supplementary Materials**

**Omics analysis of chemoresistant triple negative breast cancer cells reveals novel metabolic vulnerabilities**

**Dimitris Kordias[1,2], Christina Kostara[2#], Styliani Papadaki[2#], John Verigos[1$], Eleni Bairaktari[2] and Angeliki Magklara [1,2,3*]**

**Supplementary Figure S1. PTX-res cell line presents cross-resistance to Doxorubicin.** SUM 159 PTX-res cells treated with Doxorubicin demonstrated an increased resistance to this drug. Cell confluency was measured using the Incucyte Zoom live cell analysis system. The Doxorubicin $IC_{50}$ values of SUM 159 parental and SUM 159 PTX-res cells, were calculated using Graphpad Prism version 8.01. Data from three independent experiments performed in triplicate are shown. Error bars represent the SEM of biological replicates (n = 3).: ***: $p < 0.001$.

**Supplementary Figure S2. Bar graph showing the statistically significantly different metabolites quantified from SUM159 parental and PTX-res cells.** Extraction of metabolites using a two-phase process and metabolite quantification using the Chenomx software were conducted. Arginine, creatine phosphate, myo-inositol and phosphocholine were significantly different between the two cell lines. Error bars indicate the SEM of biological replicates (n = 6).

**Supplementary Figure S3. Metabolite enrichment analysis using MetaboAnalyst. A.** Quantitative enrichment analysis overview of the enriched metabolite sets derived from the quantified metabolites of parental and PTX-res cells based on the SMPDB library. The dashed line represents the cut-off for the significantly enriched metabolite sets in the PTX-res cells ($p<0.05$ and $FDR<0.1$). **B.** Table that summarizes the pathways that were found enriched in the PTX-res cells. Match status indicates the number of metabolites found in our data vs. the number of metabolites in the library related to a specific pathway. M; metabolism, B; biosynthesis

**Supplementary Figure S4. Typical $^1H$ NMR 500 MHz spectra of lipid extracts from SUM159 PTX-res cells and SUM159 parental cells.** The spectral region containing the signal attributed to residual water was excluded. Lipids presented in relatively higher levels in cells' membrane deflect upwards (↑ for PTX-res and ↑ for parental) and in relatively lower levels downwards (↓ for PTX-res and ↓ for parental). Abbreviations: **CE**, Cholesterol esters; **DAGPLs**, Diacyl glycerophospholipids; **DHA**,

Docosahexaenoic acid; **EPA+AA**, The sum of Eicosapentaenoic + Arachidonic Acid; **Ether GPLs**, Ether Glycerolipids; **FA**, Fatty Acids; **FC**, Free Cholesterol; **LA**, Linoleic acid; **LysoPC**, Lysophosphatidylcholine; **MeOD**, Deuterated Methanol solvent; **PL**, Phospholipids; **PC**, Phosphatidylcholine; **PE**, Phosphatidylethanolamine; **PUFA**, Polyunsaturated fatty acids; **SFA**, Saturated fatty acids; **SLs**, Sphingolipids; **SM**, Sphingomyelin; **TC**, Total Cholesterol; **TG**, Triglycerides; **UFA**. Unsaturated fatty acids *: Unknown.

**Supplementary Figure S5. Schematic representation of the cholesterol biosynthesis pathway.** The two arms of cholesterol biosynthesis pathway **(**Bloch and Kandutsch-Russell) are presented. Red shapes represent the metabolites of cholesterol biosynthesis pathway. Dashed arrows indicate multiple steps for metabolite production, while the enzymes catalyzing the steps are also presented. *MSMO1* is shown in blue.

**Table S1. The 3,184 differentially expressed genes (DEGs) between SUM159 parental and PTX resistant cells identified by RNA-sequencing.** The transcriptomes of parental and resistant cells were compared using the R package DESEQ2 and DEGs were identified by setting a 2-fold-change and a p-adj≤0.01 as cut-off values.

**Figure S1.**

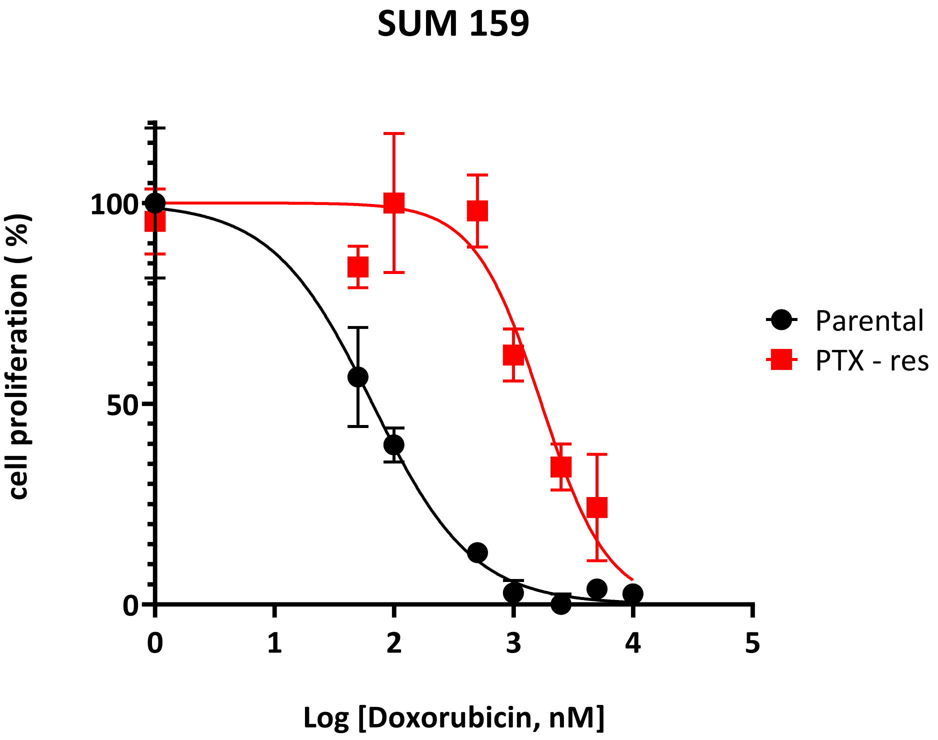


| $IC_{50}$ ( nM ) | Parental | | PTX Resistant | | |
|---|---|---|---|---|---|
| | 66.06 | ± 7.78 | 1711 | ±37.12 | p < 0.001 |

**Figure S2.**

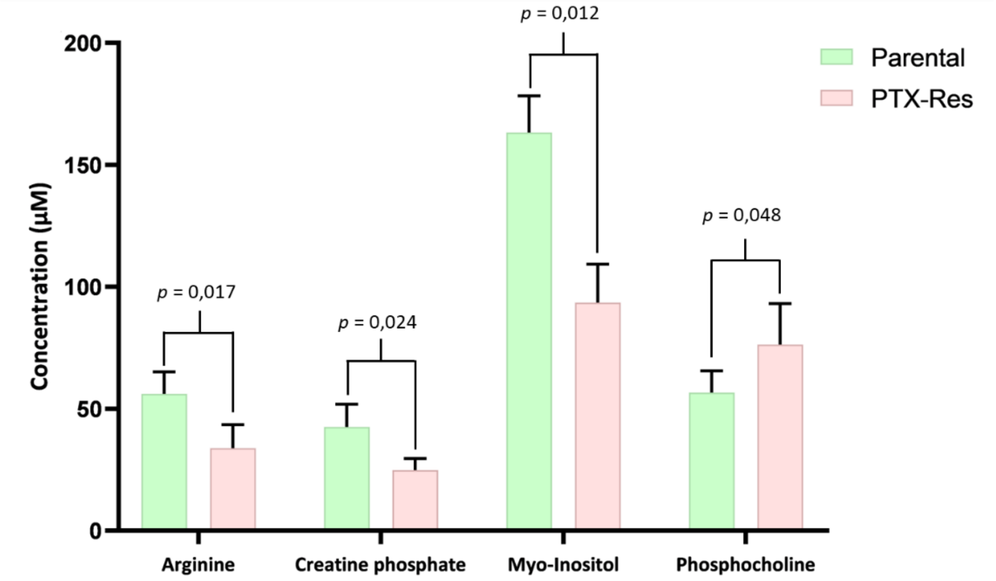

Concentration (µM)
200
150
100
50
0
p = 0,017
p = 0,024
p = 0,012
p = 0,048
Parental
PTX-Res
Arginine
Creatine phosphate
Myo-Inositol
Phosphocholine

**Figure S3.**

**A**

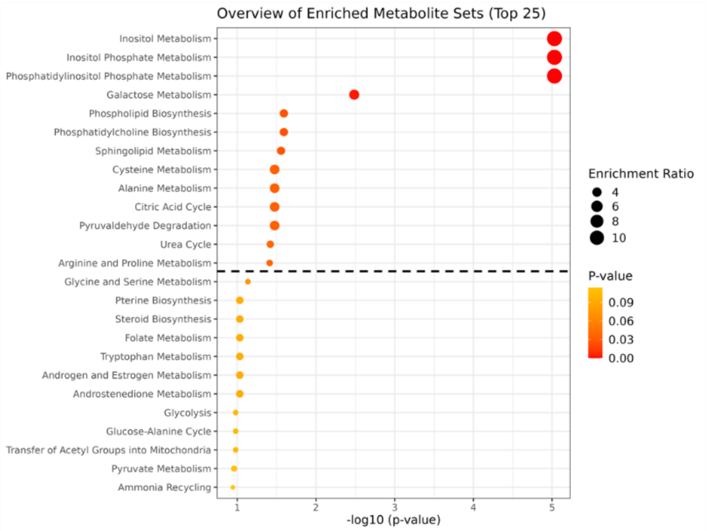


**B**

| Pathway | Match Status | p |
|---|---|---|
| Inositol M | 1/33 | 6.33E-6 |
| Inositol phosphate M | 1/26 | 9.33E-6 |
| Phosphatidylinositol signaling system | 1/17 | 9.33E-6 |
| Galactose M | 3/38 | 0.003 |
| Phospholipid B | 2/29 | 0.026 |
| Phosphatidylcholine B | 2/14 | 0.026 |
| Sphingolipid M | 2/40 | 0.028 |
| Cysteine M | 1/26 | 0.034 |
| Alanine M | 1/17 | 0.034 |
| Citric acid cycle | 1/32 | 0.034 |
| Pyruvaldehyde Degradation | 1/10 | 0.034 |
| Urea Cycle | 3/29 | 0.038 |
| Arginine and Proline M | 4/53 | 0.039 |

## Figure S4.

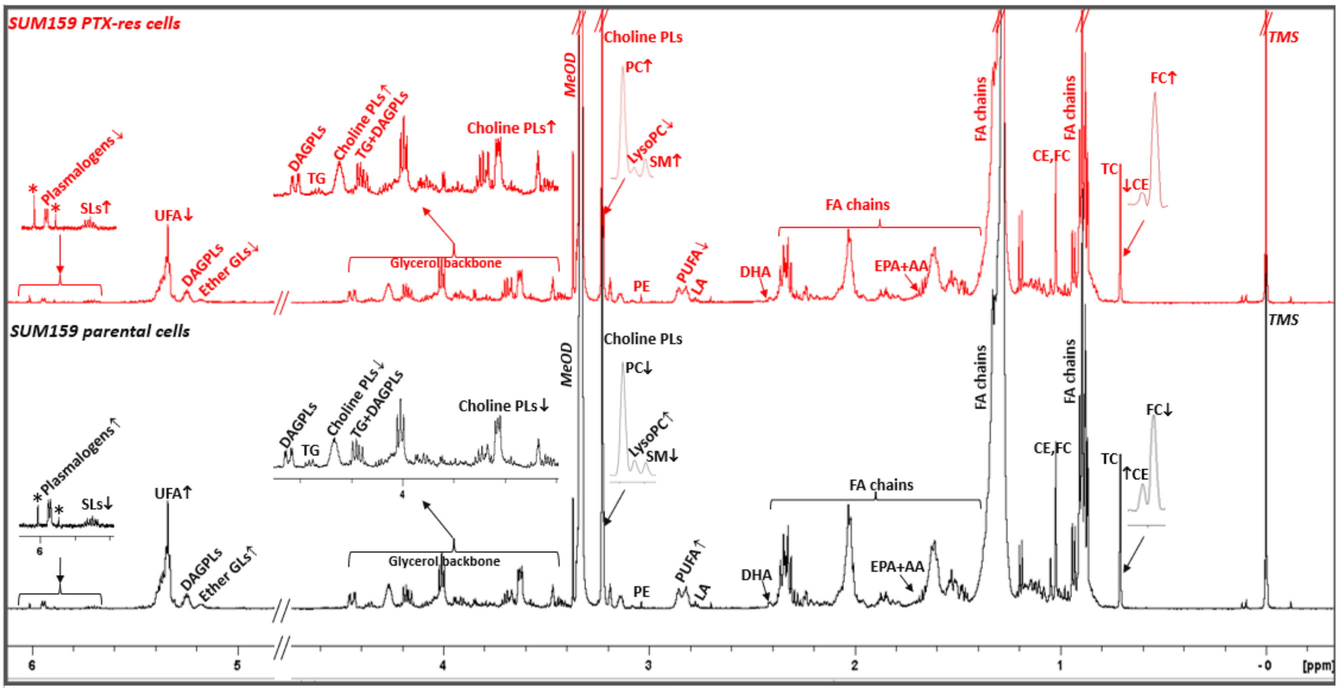
SUM159 PTX-res cells
SUM159 parental cells
MeOD
Choline PLs
PC↑
LysoPC↓
SM↑
PC↓
LysoPC↑
SM↓
TMS
FA chains
CE,FC
TC
CE
FC↑
FC↓
Plasmalogens↓
Plasmalogens↑
SLs↑
SLs↓
UFA↓
UFA↑
DAGPLs
Ether GLs↓
Ether GLs↑
TG
Choline PLs↑
Choline PLs↓
TG+DAGPLs
Glycerol backbone
PE
PUFA↓
PUFA↑
LA
DHA
EPA+AA
6
5
4
3
2
1
-0
[ppm]

Figure S5.

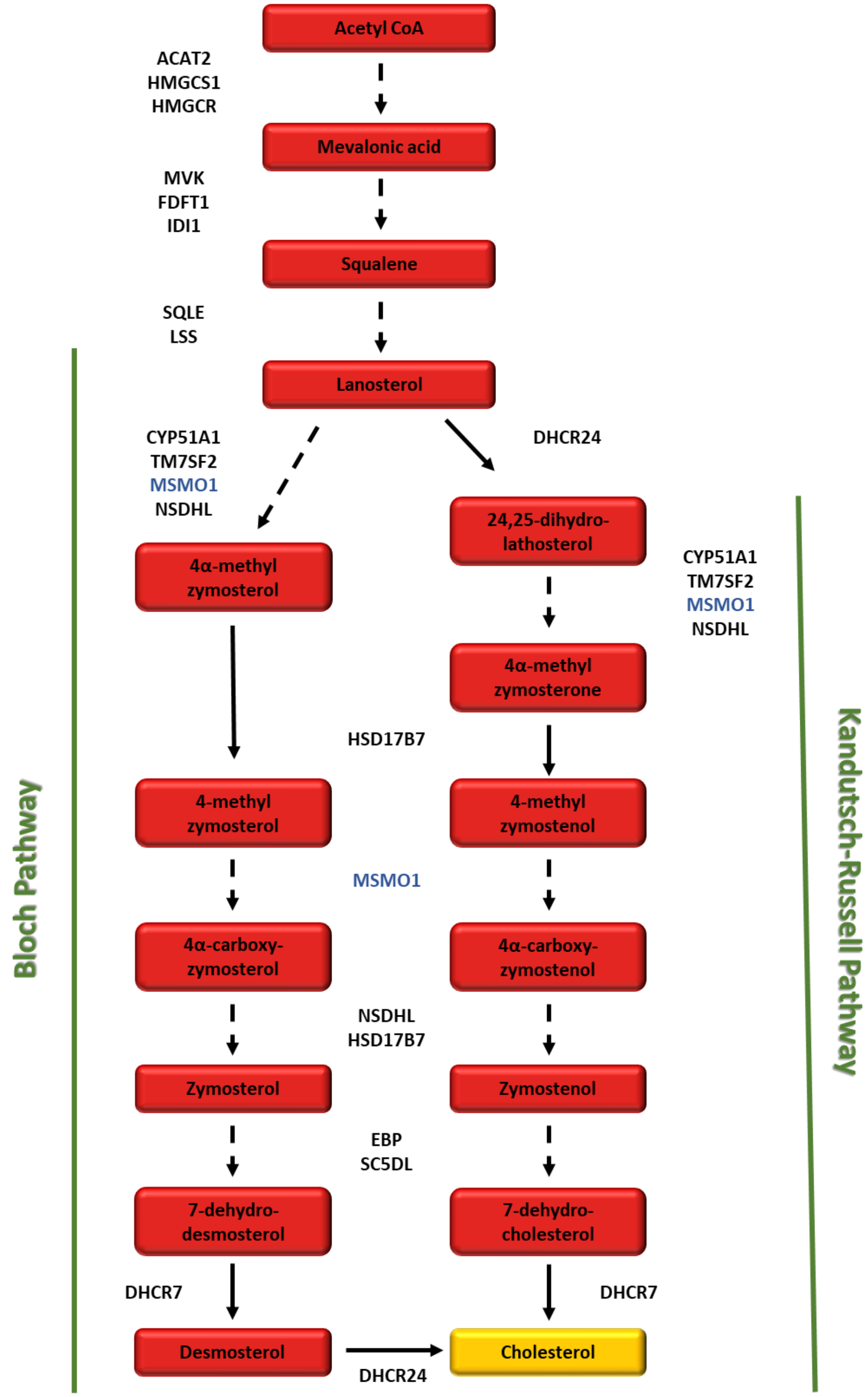
Acetyl CoA
ACAT2
HMGCS1
HMGCR
Mevalonic acid
MVK
FDFT1
IDI1
Squalene
SQLE
LSS
Lanosterol
CYP51A1
TM7SF2
MSMO1
NSDHL
DHCR24
24,25-dihydro-lathosterol
4α-methyl zymosterol
CYP51A1
TM7SF2
MSMO1
NSDHL
4α-methyl zymosterone
HSD17B7
4-methyl zymosterol
4-methyl zymostenol
MSMO1
4α-carboxy-zymosterol
4α-carboxy-zymostenol
NSDHL
HSD17B7
Zymosterol
Zymostenol
EBP
SC5DL
7-dehydro-desmosterol
7-dehydro-cholesterol
DHCR7
DHCR7
Desmosterol
Cholesterol
DHCR24
Bloch Pathway
Kandutsch-Russell Pathway

## Tables

**Table S1.** See the excel file entitled **“Suppl. Table S1”**
